# Prevalence of multi-drug resistant and extended-spectrum β-lactamase producing *Escherichia coli* and *Klebsiella pneumoniae* among meat products sold at Sohag Governorate, Egypt

**DOI:** 10.1186/s12866-025-04392-8

**Published:** 2025-10-07

**Authors:** Usama Hassan Abo-Shama, Aly El Sayed Abo-Amer, Eman Abd El-Atty Ahmed, Reem Mohamed Alsaadawy, Haitham Helmy Sayed

**Affiliations:** 1 Department of Microbiology, Faculty of Veterinary Medicine, Sohag University, Sohag, 82524 Egypt; 2 Department of Botany and Microbiology, Faculty of Science, Sohag University, Sohag, 82524 Egypt; 3 Master Researcher, Department of Microbiology, Faculty of Veterinary Medicine, Sohag University, Sohag, 82524 Egypt; 4 Department of Zoonoses, Faculty of Veterinary Medicine, Assiut University, Assiut, 71526 Egypt

**Keywords:** Egypt; *Escherichia coli*, Klebsiella pneumoniae, Meat, Multi-drug resistant, Sohag governorate

## Abstract

Meat products (MPs) are among the most commonly consumed food items in Egypt, and may serve as a potential vehicle for transmission of *Escherichia coli (E. coli)* and *Klebsiella pneumoniae (K. pneumoniae)* to humans. This study aimed to determine the prevalence of *E. coli* and *K. pneumoniae* in MPs marketed in Sohag Governorate, Egypt, with a particular focus on extended-spectrum β-lactamases (ESBL)-producing and Shiga toxin-producing *E. coli* (STEC), as well as to evaluate their antimicrobial resistance (AMR) profiles. Therefore, a total of 150 MP samples (30 of each burger, kofta, luncheon, minced meat, and sausage) were randomly collected from the stores and supermarkets in Sohag Governorate and subjected to bacteriological examinations. *E. coli* and *K. pneumoniae* were detected in 10% and 4% of the samples, respectively. Among the isolates, 66.7% of both *E. coli* and *K. pneumoniae* were multi-drug resistant (MDR), and all the isolates had a multiple antibiotic resistance (MAR) index above 0.2. Moreover, 53.3% of *E. coli* and 33.3% of *K. pneumoniae* isolates were ESBL-producers, and demonstrated higher levels of co-resistance to non-β-lactam antibiotics, compared to non-ESBL-producers isolates. PCR analysis revealed the presence of resistance and virulence genes in the investigated *E. coli* isolates (*n* = 10), including *bla*_*CTX-M*_, *bla*_*TEM*_, *dfrA*,* stx1*, and *stx2* genes, with 90%, 80%, 90%, 10%, and 20% prevalence, respectively. *E. coli* isolates carrying *stx1* or *stx2* gene were found as MDR and ESBL-producing isolates. The concordance between genotypic and phenotypic AMR ranged from 30% to 90% %, indicating varying degrees of correlation. The findings highlight the presence of MDR, ESBL-producing *E. coli*,* K. pneumoniae*, and STECs in retail MPs in Sohag Governorate, posing a potential risk to public health. These results underscore the urgent need for improved hygienic measures along the food production chain and stricter regulations on the use of antimicrobials in food animals.

**Supplementary Information:**

The online version contains supplementary material available at 10.1186/s12866-025-04392-8.

## Introduction

Meat and meat products (MPs) are one of the most nutrient-dense foods [1] and one of the most popular protein sources in the human diet [2]. MPs are popular, and customers find them more attractive than fresh meat because of their low prices, high nutritional content, exquisite flavor, quick preparation, and serving availability [3]. But unfortunately, meat and MPs could be contaminated during the slaughtering, handling, processing, preparation, and distribution [4], from the various sources [5]. Moreover, they can be a beneficial environment for these microorganisms’ growth [6]. The quality of meat and MPs is lost because of microbial contamination, which also represents a risk to public health [7] and has significant economic consequences [8].

Meat and MPs are considered to be the main sources of foodborne pathogens (FBPs), which are the primary causes of infection and death in developing nations [9]. To provide safe food and prevent foodborne diseases, early detection of FBPs is necessary [10]. The use of indicator bacteria that indicate the food product’s safety status is encouraged due to the difficulties of monitoring and detecting all FBPs present in the product to assess its safety [11]. According to Edris et al. [12], food safety authorities consider *Enterobacteriaceae* and/or their members to be a reliable microbiological indicator of food safety, quality, and hygiene.

Family *Enterobacteriaceae* includes a wide variety of Gram-negative rod species that are found naturally in the gastrointestinal system of animals as well as in other environments [12]. *Escherichia coli (E. coli)* has emerged as a dangerous FBP linked to numerous outbreaks [13]. According to Lee et al. [14], it is currently the most common pathogen found in meat and MPs, and it has caused multiple outbreaks through these foods [15]. Enteric *E. coli* is classified into six primary pathotypes based on specific virulence factors and pathogenic characteristics [16]. The most toxic pathotype is Shiga toxin-producing *E. coli* (STEC**)** as a zoonotic pathogen [17], and meat and its products are a major source for its transmission to humans [18]. It causes serious disorders in humans as bloody diarrhea, hemorrhagic colitis, thrombotic thrombocytopenic purpura, and the deadly hemolytic-uremic syndrome [19, 20]. Shiga toxin production, which prevents the host cells from synthesizing proteins and ultimately results in their death, is the primary characteristic of STEC [21]. Human pathogenicity is closely correlated with Shiga toxins, which are considered to be the primary virulence factors of STEC strains [22].

*Klebsiella pneumoniae (K. pneumoniae)* is an important opportunistic pathogen that can cause a variety of disorders in humans, including pneumonia and sepsis, particularly in young children, the elderly, and people with compromised immune systems. Furthermore, it is now a major nosocomial pathogen [23]. *K. pneumoniae* is a common FBP, and more foodborne outbreaks have been recorded in various countries recently [24]. According to Deepan et al. [25], it is isolated from meat and MPs, which may be the source of infection for humans. Gastrointestinal *K. pneumoniae* carriage is believed to be a risk factor for liver abscess in several Asian nations, this correlation was less prevalent outside of Asia [26].

Antimicrobial resistance (AMR) remains a persistent global problem [27]. β-lactams are one of the most significant groups of antibiotics [28]. Since they have a high potential for killing both Gram-positive and Gram-negative bacteria with minimum side effects, they are widely used to treat a variety of infections. Unfortunately, a lot of bacterial species resist almost all β-lactams by producing extended-spectrum β-lactamases **(**ESBLs), which are efficient hydrolyzers of β-lactams [29]. Additionally, multi-drug resistance and resistance to additional antimicrobials (AMs) are frequently found in ESBL-producing bacteria, which makes treatment more difficult, prolongs sickness, raises treatment costs, and increases the possibility of therapy failure [30].

WHO has classified ESBL-producing *Enterobacteriaceae* as a critical priority pathogen [25], and they are an emerging problem [31]. ESBLs production by *Enterobacteriaceae* has increased in recent years. According to Raswan et al. [31], ESBL-encoding genes are prevalent in *E. coli* and *K. pneumoniae*, showing a high rate of multidrug resistance. Additionally, the number of infections caused by ESBL-producing *Enterobacteriaceae* has increased [29], and ESBL-producing *E. coli* and *K. pneumoniae* have been the main species associated with nosocomial infections [27]. Environment (soil and water), livestock, food, wildlife, and pets are considered reservoirs for ESBL-producing *E. coli* and *K. pneumoniae* [32]. Consequently, foods may act as a vehicle for the transfer of these stains to the consumer’s gastrointestinal tract [33] where they can transfer antimicrobial resistance genes (AMR genes) to other pathogens [26, 28].

This is the first study on ESBL-producing *E. coli* and *K. pneumoniae* at Sohag Governorate, Egypt, and to the best of our knowledge, there are limited studies on the prevalence of *E. coli*, *K. pneumoniae*, and STEC among MPs provided there and their AMR. These data are essential for determining MPs’ role in the potential hazards to public health and the adverse impacts for these FBPs may have on the economy, as well as recognizing the potential problems that may occur during the production and distribution of these products, accordingly creating efficient intervention strategies for preventive and control measures. Thus, the purpose of this study was to determine the prevalence of *E. coli* and *K. pneumoniae* in MPs (burger, kofta, luncheon, minced meat, and sausages) sold in Sohag Governorate, Egypt, particularly those producing ESBL as well as STEC, and to assess their AMR profiles.

## Materials and methods

### Study area and sampling

**Fig. 1:**
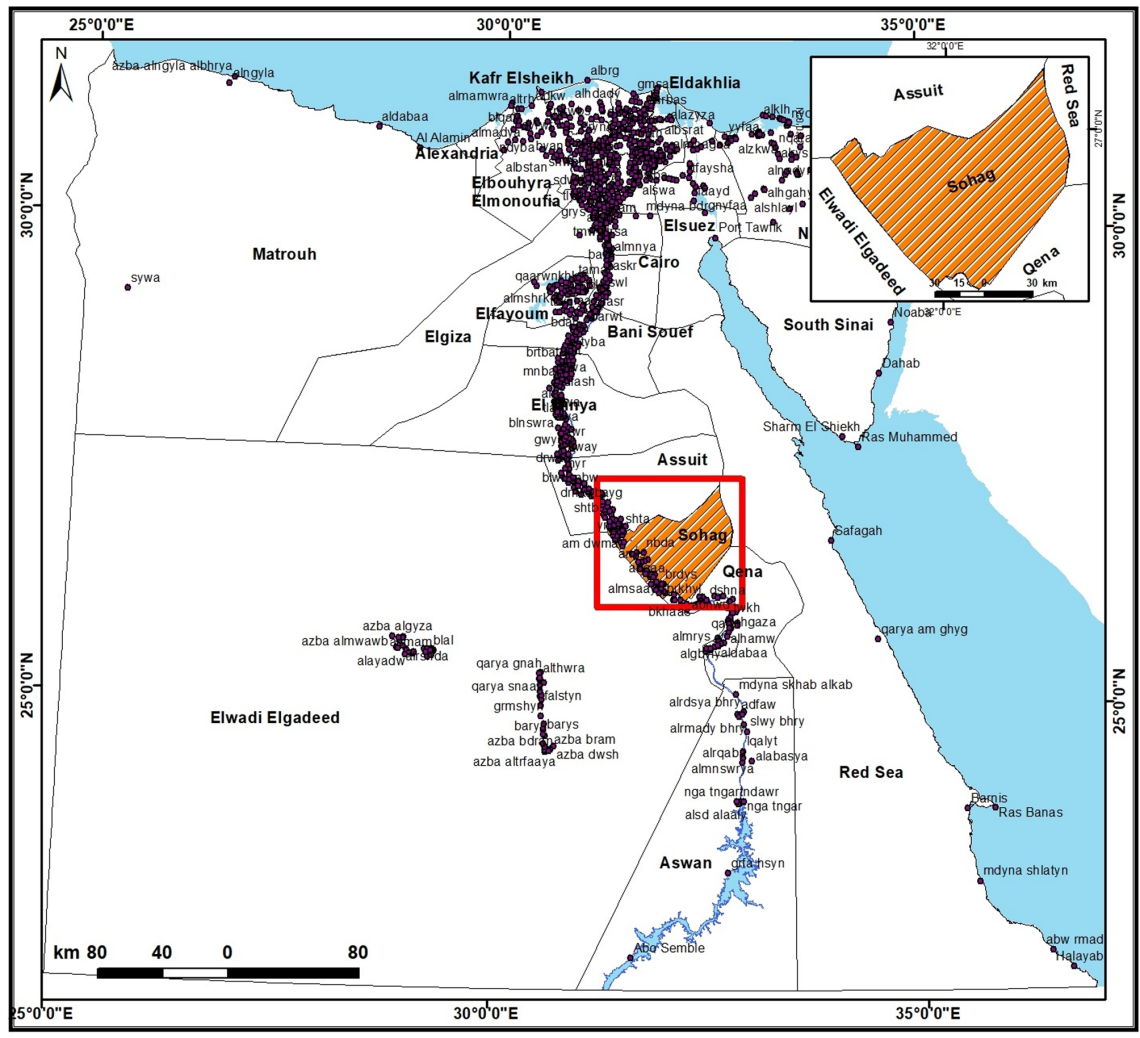
An illustration showing the map of Egypt with the study area marked

A total of 150 samples of MPs, including burgers, kofta, luncheon, minced meat, and sausage (30 of each), were randomly collected from the different stores and supermarkets at Sohag Governorate, Egypt **(**Fig. 1**)**, during the period from July 2023 to April 2024. Each sample was packed in a sterile plastic bag and transferred immediately to the laboratory in an insulated ice box for bacteriological examination.

### Preparation of the samples

Once the frozen samples had been thawed, 25 g of each analyzed sample were mixed with 225 ml of buffered peptone water 0.1% (Oxoid, UK) and homogenized thoroughly with a blender. Pre-enrichment was subsequently carried out aerobically at 37 °C for 24 h [34].

### Isolation and identification of E. coli and K. pneumoniae

A loopful of each enriched broth was streaked on MacConkey agar (Himedia Laboratories, India) and incubated aerobically at 37 °C for 24–48 h. Following that, pink colonies isolated on MacConkey agar were streaked over Eosin Methylene Blue Agar (Oxoid, UK) and incubated aerobically at 37 °C for 24–48 h [8, 35].

Later, suspicious green colonies with a metallic sheen and pink mucoid colonies were selected and inspected under a microscope after Gram staining and tested for oxidase and catalase. Finally, following the manufacturer’s instructions, the Vitek 2 system (BioMérieux, France) was used to identify the isolates that were Gram-negative bacilli, oxidase-negative, and catalase-positive. Until they were required again, isolates were kept at −80 °C in tryptone soy broth (TSB) (Oxoid, UK) with 15% glycerol (El Naser Co., Egypt) [16].

### Antimicrobial susceptibility testing for E. coli and K. pneumoniae isolates

Antimicrobial susceptibility and ESBL production of *E. coli* and *K. pneumoniae* isolates were determined by the Vitek 2 system using Vitek 2 AST-GN73 (BioMérieux, France). Cards were inoculated and incubated in the Vitek 2 system according to the manufacturer’s instructions, and the results were interpreted by using the Advanced Expert System (AES). *E. coli* or *K. pneumoniae* isolate was considered multi-drug resistant (MDR) when it was resistant to three AMs of different classes or more, and the multiple antibiotic resistance (MAR) index was calculated for each isolate by dividing the number of AMs to which the isolate was resistant by the number of the tested AMs [36].

### Detection of some antimicrobial resistance and Shiga toxin genes in E. coli isolates by PCR

Due to the limited funds, only 10 randomly selected *E. coli* isolates were investigated by PCR for the presence of some AMR genes (*bla*_*CTX−M*_, *bla*_*TEM*,_ and *dfrA*) and Shiga toxin genes (*stx1* and *stx2*). Four positive controls were kindly provided by Reference Laboratory for Veterinary Quality Control on Poultry Production, Animal Health Research Institute, Giza, Egypt, including *E. coli* NCTC 13,353 for *bla*_*CTX−M*_ gene, *E. coli* ATCC 35,218 for *bla*_*TEM*_ gene, *E. coli* ATCC 43,894 for both *stx1* and *stx2* genes, as well as a field *K. pneumoniae* isolate was previously confirmed to be positive for *dfrA* gene by PCR and sequencing. Nuclease-free water was used as a negative control.

Freshly grown *E. coli* in TSB (Oxoid, UK) were collected, and DNA was extracted by using QIAamp DNA Mini Kit (Qiagen GmbH, Germany) according to the manufacturer’s instructions.

PCR was performed for the detection of the targeted genes by using the oligonucleotide primers (Metabion, Germany) illustrated in Table 1 and EmeraldAmp GT PCR Master Mix (Takara, Japan). The reaction mixture was prepared in a total volume of 25 µl according manufacturer’s instructions, consisting of Master Mix (12.5 µl), extracted DNA (5 µl), forward primer (1 µl), reverse primer (1 µl), and nuclease-free water (5.5 µl). DNA Amplification was performed in a T3 thermocycler (Biometra, Germany) under PCR conditions conditions as described by Monstein [37], Dallenne [38], Grape [39], and Gannon [40] for *bla*_*CTX−M*_, *bla*_*TEM*_, *dfrA*, as well as *stx1* and *stx2* genes, respectively.

**Table 1:** Target genes of *E. coli* and their oligonucleotide primers used in the study

Target gene	Primers sequences (5`- 3`)	Product size (bp)	Reference
*stx1*	ACACTGGATGATCTCAGTGG	614	[40]
	CTGAATCCCCCTCCATTATG		
*stx2*	CCATGACAACGGACAGCAGTT	779	
	CCTGTCAACTGAGCAGCACTTTG		
*bla*_CTX−M_	ATGTGCAGYACCAGTAARGTKATGGC	593	[37]
	TGGGTRAARTARGTSACCAGAAYCAGCGG		
bla_TEM_	CATTTCCGTGTCGCCCTTATTC	800	[38]
	CGTTCATCCATAGTTGCCTGAC		
dfrA	TGGTAGCTATATCGAAGAATGGAGT	425	[39]
	TATGTTAGAGGCGAAGTCTTGGGTA		

PCR products and a 100 bp DNA ladder (Thermo Fishers Scientific, Lithuania) were electrophoresed through a 1.5% agarose gel (Biometra, Germany), the gel was photographed by a gel documentation system (Alpha Innotech, USA), and the data were analyzed.

## Results

###  Prevalence of E. coli and K. pneumoniae among the examined meat product samples

Based on the morphological and biochemical characters of the bacterial isolates, *E. coli* and *K*. *pneumoniae* were isolated from 15 (E1-E15) and 6 (K1-K6) samples of the examined samples, with a total prevalence of 10% and 4%, respectively. The prevalence of *E. coli* and *K*. *pneumoniae* among the different examined MPs is illustrated in Table 2.

**Table 2:** Prevalence of *E. coli* and *K*. *pneumoniae* among the examined meat products

Meat product	E. coli		K. pneumoniae	
	No.	%	No.	%
Burger (*n* = 30)	2	6.7	1	**3.3**
Kofta (*n* = 30)	5	16.7	0	**0**
Luncheon (*n* = 30)	4	13.3	3	**10**
Minced meat (*n* = 30)	3	10	0	**0**
Sausage (*n* = 30)	1	3.3	2	**6.7**
Total (*n* = 150)	**15**	**10**	**6**	**4**

###  Antimicrobial susceptibility of E. coli and K. pneumoniae isolates

Vitek 2 system revealed that *E. coli* isolates were resistant to AMP, CFZ, CIP, CRO, FOX, SAM, SXT, and TZP (Table 3) while *K. pneumoniae* isolates were resistant to AMK, AMP, CAZ, CFZ, CIP, CRO, FEP, FOX, GEN, SAM, SXT, and TZP (Table 4).

**Table 3:** Results of antimicrobial susceptibility of *E. coli* isolates by Vitek 2 system

Antimicrobial	MIC Calling Range	Results (*n* = 15)					
		S.		I.		*R*.	
		No.	%	No.	%	No.	%
Ampicillin (AMP)	2–32	0	0	0	0	15	100
Ampicillin/Sulbactam (SAM)	2–32	4	26.7	0	0	11	73.3
Piperacilllin/Tazobactam (TZP)	4–128	5	33.3	0	0	10	66.7
Cefazolin (CFZ)	4–64	2	13.3	1	6.7	12	80
Cefoxitin (FOX)	4–64	4	26.7	0	0	11	73.3
Ceftazidime (CAZ)	1–64	8	53.3	0	0	7	46.7
Ceftriaxone (CRO)	1–64	4	26.7	0	0	11	73.3
Cefepime (FEP)	1–64	9	60	0	0	6	40
Meropenem (MEM)	0.25–16	8	53.3	0	0	7	46.7
Amikacin (AMK)	2–64	11	73.3	1	6.7	3	20
Gentamicin (GEN)	1–16	10	66.7	2	13.3	3	20
Tobramycin (TOB)	1–16	8	53.3	0	0	7	46.7
Ciprofloxacin (CIP)	0.25–4	7	46.7	0	0	8	53.3
Levofloxacin (LVX)	0.12–8	7	46.7	1	6.7	7	46.7
Nitrofurantoin (NIT)	16–512	12	80	2	13.3	1	6.7
Trimethoprim/Sulfamethoxazole (SXT)	20–320	3	20	1	6.7	11	73.3

**Table 4:** Results of antimicrobial susceptibility of *K*. *pneumoniae* isolates by Vitek 2 system

Antimicrobial	MIC Calling Range	Results (*n* = 6)					
		S.		I.		*R*.	
		No.	%	No.	%	No.	%
Ampicillin (AMP)	2–32	0	0	0	0	6	100
Ampicillin/Sulbactam (SAM)	2–32	0	0	0	0	6	100
Piperacilllin/Tazobactam (TZP)	4–128	0	0	2	33.3	4	66.7
Cefazolin (CFZ)	4–64	0	0	0	0	6	100
Cefoxitin (FOX)	4–64	0	0	2	33.3	4	66.7
Ceftazidime (CAZ)	1–64	1	16.7	0	0	5	83.3
Ceftriaxone (CRO)	1–64	1	16.7	2	33.3	3	50
Cefepime (FEP)	1–64	2	33.3	0	0	4	66.7
Meropenem (MEM)	0.25–16	4	66.7	0	0	2	33.3
Amikacin (AMK)	2–64	3	50	0	0	3	50
Gentamicin (GEN)	1–16	3	50	0	0	3	50
Tobramycin (TOB)	1–16	5	83.3	0	0	1	16.7
Ciprofloxacin (CIP)	0.25–4	3	50	0	0	3	50
Levofloxacin (LVX)	0.12–8	2	33.3	2	33.3	2	33.3
Nitrofurantoin (NIT)	16–512	1	16.7	3	50	2	33.3
Trimethoprim/Sulfamethoxazole (SXT)	20–320	0	0	0	0	6	100

It also revealed that 53.3% and 33.3% of *E. coli* and *K. pneumoniae* isolates were ESBL-producers, respectively. Furthermore, it was found that 66.7% of each *E. coli* and *K. pneumoniae* isolates were MDR, and all the bacterial isolates had a MAR index of more than 0.2, as illustrated in Table 5.

**Table 5:** Antimicrobial resistance profiles of *E. coli* and *K. pneumoniae* isolates

Isolate No.	Antimicrobial resistance pattern	ESBL	Isolates of this pattern		Number of resistant		MDR	MAR Index
			No.	%	AMs	AMs classes		
E1, 8 & 9	AMP, SAM, TZP, CFZ, FOX, CAZ, CRO, FEP, MPM, TOB, CIP, LVX, SXT.	+	3	20	13	4	MDR	**0.813**
E6	AMP, SAM, TZP, FOX, CAZ, CRO, FEP, MPM, TOB, CIP, LVX, SXT.	+	1	6.7	12	4	MDR	**0.750**
E10	AMP, SAM, TZP, CFZ, FOX, CAZ, CRO, FEP, MPM, CIP, LVX, SXT	+	1	6.7	12	3	MDR	**0.750**
E4	AMP, SAM, TZP, CFZ, FOX, CAZ, CRO, CIP, SXT.	-	1	6.7	9	3	MDR	**0.563**
E13	AMP, SAM, TZP, CFZ, CRO, MPM, CIP, LVX, SXT.	+	1	6.7	9	3	MDR	**0.563**
E5	AMP, SAM, CFZ, FOX, AMK, GEN, TOB, SXT.	+	1	6.7	8	3	MDR	**0.500**
E11	AMP, TZP, CRO, MPM, CIP, LVX, SXT.	-	1	6.7	7	3	MDR	**0.438**
E15	AMP, TZP, CRO, GEN, TOB, NIT.	-	1	6.7	6	3	MDR	**0.375**
E2	AMP, SAM, CFZ, FOX, AMK, TOB.	-	1	6.7	6	2	Not	**0.375**
E3	AMP, SAM, CFZ, FOX, AMK, GEN.	-	1	6.7	6	2	Not	**0.375**
E12	AMP, TZP, CFZ, FOX, CRO, SXT.	-	1	6.7	6	2	Not	**0.375**
E7	AMP, SAM, CFZ, FOX, SXT.	-	1	6.7	5	2	Not	**0.313**
E14	AMP, CFZ, CAZ, CRO, FEP.	+	1	6.7	5	1	Not	**0.313**
Total (%) for *E. coli *isolates (*n* = 15)		**8 (53.3%)**	15	100			**10 (66.7%)**	
K1	AMP, SAM, TZP, CFZ, FOX, CAZ, CRO, FEP, MPM, AMK, GEN, TOB, CIP, LVX, NIT, SXT	+	1	16.7	16	5	MDR	**1.000**
K6	AMP, SAM, TZP, CFZ, FOX, CRO, FEP, MPM, AMK, GEN, CIP, LVX, SXT.	-	1	16.7	13	4	MDR	**0.813**
K2	AMP, SAM, TZP, CFZ, FOX, CAZ, FEP, AMK, GEN, NIT, SXT	+	1	16.7	11	4	MDR	**0.688**
K3	AMP, SAM, TZP, CFZ, FOX, CAZ, SXT.	-	1	16.7	7	2	Not	**0.438**
K5	AMP, SAM, CFZ, CAZ, CRO, FEP, SXT.	-	1	16.7	7	2	Not	**0.438**
K4	AMP, SAM, CFZ, CAZ, CIP, SXT.	-	1	16.7	6	3	MDR	**0.375**
Total (%) for *K. pneumoniae* isolates (*n* = 6)		**2 (33.3%)**	**6**	**100**			**4 (66.7%)**	

On the other hand, comparison of AMR of ESBL and non-ESBL-producing isolates to non-β-lactam AMs revealed that ESBL-producing isolates were more resistant to non-β-lactam AMs than non-ESBL-producing isolates. Co-resistance of ESBL and non-ESBL-producing *E. coli* and *K. pneumoniae* isolates to non-β-lactam AMs was illustrated in Table 6. Also, it was found that 90% of ESBL-producing isolates (*n* = 9 of 10) were MDR, comparable to only 45.5% of MDR isolates among non-ESBL-producing isolates (*n* = 5 of 11).

**Table 6:** Co-resistance of ESBL and non-ESBL-producing isolates to non-β-lactam antimicrobials

Antimicrobial	*E. coli* (*n* = 15)				*K*. *pneumoniae* (*n* = 6)				Total (*n* = 21)			
	ESBL (+) (*n* = 8)		ESBL (-) (*n* = 7)		ESBL (+) (*n* = 2)		ESBL (-) (*n* = 4)		ESBL (+) (*n* = 10)		ESBL (-) (*n* = 11)	
	No.	%	No.	%	No.	%	No.	%	No.	%	No.	%
Amikacin (AMK)	1	12.5	2	28.6	2	100	1	25	3	30	3	**27.3**
Gentamicin (GEN)	1	12.5	2	28.6	2	100	1	25	3	30	3	**27.3**
Tobramycin (TOB)	5	62.5	2	28.6	1	50	0	0	6	60	2	**18.2**
Ciprofloxacin (CIP)	6	75.0	2	28.6	1	50	2	50	7	70	4	**36.4**
Levofloxacin (LVX)	6	75.0	1	14.3	1	50	1	25	7	70	2	**18.2**
Nitrofurantoin (NIT)	0	0	1	14.3	2	100	0	0	2	20	1	**9.1**
Trimethoprim/Sulphamethoxazole (SXT)	**7**	**87.5**	**4**	**57.1**	**2**	**100**	**4**	**100**	**9**	**90**	**8**	**72.7**

### Incidence of antimicrobial resistance and Shiga toxin genes among the investigated E. coli isolates

As illustrated in Supplementary Figs. 1–5 and Table 7, 90%, 80%, 90%, 10%, and 20% of the investigated *E. coli* isolates by PCR (*n* = 10), harbored *bla*_*CTX−M*_, *bla*_*TEM*,_ *dfrA*,* stx1* and *stx2* genes, respectively, and various combinations from these genes were found in these isolates. *Bla*_*CTX−M*_ and *bla*_*TEM*_ genes were found together in 70% of the investigated isolates and associated with *dfrA* in 60% of them. Also, *E. coli* isolates carrying *the stx1* or *stx2* gene harbored all the investigated AMR genes or two of them at least, and they were ESBL producers and MDR.

**Table 7:** Phenotypic and genotypic characteristics for *E. coli* isolates investigated by PCR (*n* = 10)

Isolate No.	Isolate origin	Phenotypic antimicrobial resistance													Genotypic antimicrobial resistance			Shiga toxin genes	
		AMP	SAM	TZP	CFZ	FOX	CAZ	CRO	FEP	MPM	SXT	ESBL		MDR	bla_CTX−M_	bla_TEM_	dfrA	stx1	stx2
E1	Minced meat	R	R	R	R	R	R	R	R	R	R	+	MDR		-	+	+	-	**+**
E2	Burger	R	R	S	R	R	S	S	S	S	S	-	Not		+	+	+	-	**-**
E4	Kofta	R	R	R	R	R	R	R	S	S	R	-	MDR		+	+	+	-	**-**
E5	Sausage	R	R	S	R	R	S	S	S	S	R	+	MDR		+	+	+	-	**-**
E7	Burger	R	R	S	R	R	S	S	S	S	R	-	Not		+	+	+	-	**-**
E8	Luncheon	R	R	R	R	R	R	R	R	R	R	+	MDR		+	+	-	-	**-**
E10	Kofta	R	R	R	R	R	R	R	R	R	R	+	MDR		+	+	+	-	**+**
E11	Luncheon	R	S	R	I	S	S	R	S	R	R	**-**	MDR		+	+	+	-	**-**
E13	Minced meat	R	R	R	R	S	S	R	S	R	R	+	MDR		+	-	+	+	**-**
E14	Kofta	R	S	S	R	S	R	R	R	S	S	+	Not		+	-	+	-	**-**
Total (%)		**100**	**80**	**60**	**90**	**70**	**50**	**70**	**40**	**50**	**80**	**60**	**70**		**90**	**80**	**90**	**10**	**20**

On the other hand, it was found that the coincidence rate between phenotypic and genotypic AMR ranged from 30% to 90%, as illustrated in Table 8.

**Table 8:** Coincidence rate of phenotypic and genotypic antimicrobial resistance in *E*. *coli* isolates (*n* = 10)

Antimicrobial agent/ESBL confirmation test	Antimicrobial resistance gene	No. of isolates (%)
Ampicillin (AMP)	*bla*_*CTX−M*_	9/10 (90%)
Ampicillin/Sulbactam (SAM)	*bla*_*CTX−M*_	7/10 (70%)
Piperacilllin/Tazobactam (TZP)	*bla*_*CTX−M*_	5/10 (50%)
Cefazolin (CFZ)	*bla*_*CTX−M*_	8/10 (80%)
Cefoxitin (FOX)	*bla*_*CTX−M*_	6/10 (60%)
Ceftazidime (CAZ)	*bla*_*CTX−M*_	4/10 (40%)
Ceftriaxone (CRO)	*bla*_*CTX−M*_	6/10 (60%)
Cefepime (FEP)	*bla*_*CTX−M*_	3/10 (30%)
Meropenem (MEM)	*bla*_*CTX−M*_	4/10 (40%)
ESBL confirmation test	*bla*_*CTX−M*_	5/10 (50%)
Ampicillin (AMP)	*bla*_*TEM*_	8/10 (80%)
Ampicillin/Sulbactam (SAM)	*bla*_*TEM*_	7/10 (70%)
Piperacilllin/Tazobactam (TZP)	*bla*_*TEM*_	5/10 (50%)
Cefazolin (CFZ)	*bla*_*TEM*_	7/10 (70%)
Cefoxitin (FOX)	*bla*_*TEM*_	7/10 (70%)
Ceftazidime (CAZ)	*bla*_*TEM*_	4/10 (40%)
Ceftriaxone (CRO)	*bla*_*TEM*_	5/10 (50%)
Cefepime (FEP)	*bla*_*TEM*_	3/10 (30%)
Meropenem (MEM)	*bla*_*TEM*_	4/10 (40%)
ESBL confirmation test	*bla*_*TEM*_	4/10 (40%)
Trimethoprim/Sulfamethoxazole (SXT)	*dfrA*	7/10 (70%)

## Discussion

Meat and MPs contamination may result in quality deterioration and public health problems [7]. Members of the *Enterobacteriaceae* family are the most challenging and prevalent bacterial contaminants detected in meat and MPs worldwide [41], and they have epidemiological interest and importance [7]. With a focus on MDR and ESBL-producing strains as well as STEC, this paper discusses the hazard posed by *E. coli* and *K. pneumoniae* present in MPs sold in Sohag Governorate, Egypt, and their AMR.

In the present study, the prevalence of *E. coli* among the investigated MPs samples was in agreement with the results of Gamal et al. [42]. who reported that the prevalence of *E. coli* among MPs sold in Kaliobia Governorate, Egypt, was 10.5%, while much higher prevalences of *E. coli* were reported by Mohammed et al. [20] and Sallam et al. [43] among MPs sold at Mansoura city, Egypt. On the other hand, the prevalence of *K. pneumoniae* among the examined samples was consistent with findings from El Gendy et al. [44] that *K. pneumoniae* was present in 4% of MPs sold in Alexandria Governorate, Egypt, while Elhawary et al. [7] and EL Bayoumi et al. [6] found that *K. pneumoniae* was present in 11.3% and 24% of MPs sold in Assiut and Gharbia Governorates, Egypt, respectively. These variations in the prevalence of *E. coli* and *K. pneumoniae* could be due to the differences in handling and hygienic practices during the manufacturing stages [28] as well as geographic location [36]. Furthermore, as illustrated in Table 2, E. *coli* and *K*. *pneumoniae* were isolated from the different MPs with variable percentages and this could be attributed to the differences in the handling method of each product, processing operations number to which each product was subjected, the post-processing contamination amount and storage conditions [45].

In addition to *K. pneumoniae* isolated from luncheon and sausage samples, the high incidence of *E. coli* reported in most of the MPs investigated in this study may be due to inadequate manufacturing and distribution procedures [15] as well as improper storage conditions [41]. Additionally, this indicates fecal contamination with the possibility of the presence of other enteric pathogens in the examined MP samples [17], which could be a public health concern. As a result, strict observance of hygienic and food handling procedures must be conducted from farm to fork, and food handlers must be fully aware of these procedures.

The pathogenic bacteria’s virulence potential is determined by their virulence genes [46]. STEC’s capacity to cause fatal diseases in humans has been associated with the production of *stx1*,* stx2*, or both [47]. Surveys have shown that E. coli strains harboring the stx2 gene are potentially more virulent than those harboring the stx1 [20]. According to this study, 10% and 20% of the isolates under investigation carried the *stx1* and *stx2* genes, respectively, and were MDR and ESBL-producing isolates. These findings indicate that MPs were contaminated with MDR ESBL-producing STEC and that consumers are more likely to contract severe foodborne infection consequences. Our findings were almost in concurrence with those of Ezzat et al. [9] and Mohammed [5], who reported that the *stx1* gene was present in 4.3% and 6.3% of *E. coli* isolated from MPs, respectively. They also concurred with Ezzat et al. [9], who reported that the *stx2* gene was present in 17.4% of *E. coli* isolated from MPs. However, El-Bagory et al. [3, 22] reported that the *stx1* gene was present in 50% and 58.1% of the *E. coli* isolated from MPs, respectively, and the *stx2* gene was present in 83.3% and 74.2% of them, respectively. Also, Mohammed et al. [20] found that 46.7% and 66.7% of non-O157 STEC isolated from MPs were positive for *stx1* and *stx2* genes, respectively.

AMR increase in *Enterobacteriaceae* members has become a main threat to public health [48], and the MDR phenomenon is becoming particularly prominent in *E. coli* and *K. pneumoniae* [8]. Regarding AMR of *E. coli* isolates in this study, the results were opposed to those of Hassanien et al. [45] and Gweshe et al. [17]. for all the tested AMs, while Gamal et al. [42], Abdel-Atty et al. [28], and Ronald et al. [8]. had similar resistance parentages for GEN, CIP, and SXT, respectively. Additionally, they corroborated the findings of Ronald et al. [8], who found that 70% of *E. coli* isolates were MDR, whereas Abdel-Atty et al. [28] and Elsherbeny et al. [36] found that 100.0% and 13.3% of *E. coli* isolates were MDR.

On the other hand, similar resistance percentages for *K. pneumoniae* isolates to AMP (100%), TZP (69.2%), and CAZ (92.3%) were reported by Madhup et al. [49]. However, Gobarah et al. [35] reported that isolates of *K. pneumoniae* that were sensitive to FEP, CRO, CAZ, SXT, and AMP were 100%, 85.7%, 71.4%, 57.2%, and 14.3%, respectively. Furthermore, our findings were in close concurrence with those of Nirwati et al. [50], who reported that 54.5% of *K. pneumoniae* isolates were MDR, whereas Ammar et al. [23] and Elsherbeny et al. [36] revealed that 90.9% and 16.6% of *K. pneumoniae* isolates were MDR, respectively. The variations in AMR of our isolates and the different studies could be attributed to the differences in AMs usage levels in the different regions [8], type of tested food samples [25], number of tested samples, and**/**or to methodological heterogeneity [31].

Nevertheless, the high levels of AMR and multi-drug resistance observed in our isolates of *E. coli* and *K. pneumoniae* may be due to the improper use of AMs in Egypt in both humans and animals, as well as the transfer of AMR genes among the various bacteria [23], which are the main factors of the widespread spread of AMR [46]. Also, the restricted availability and usage of some of these AMs in Egyptian cattle and buffaloes are comparable to the excessive and continuous use of the other AMs, such as AMP and SXT, which may account for the isolates’ low resistance against NIT, AMK, GEN, TOB, LEV, MEM, and FEP (Tables 3 and 4).

MAR index is used as a valuable tool for the assessment of the health risks associated with AMR in bacteria. A MAR index value ≥ 0.2 suggests that bacteria probably originate from environments where AMs are frequently used or have been previously exposed to AMs, posing a significant risk [8]. In this study, all the bacterial isolates had a MAR index exceeding 0.2, so they pose high health risks to MPs consumers. Therefore, indiscriminate use of AMs must be prohibited, and monitoring AMR of the pathogenic bacteria present in MPs should be strengthened.

ESBL production is considered the prevalent mechanism for β-lactams resistance in Gram-negative bacteria [35] and represents a significant public health concern [30]. According to our results, there was a high prevalence of ESBL-producing *E. coli* and *K. pneumoniae* among the investigated MPs, in agreement with the results of Ahmed et al. [51], who reported that 58% of *E. coli* isolates were ESBL-producers, while Rashwan et al. [31] found that 66% of *E. coli* isolates were ESBL-producers. Moreover, 33.3% of *K. pneumoniae* isolates were ESBL-producers, in agreement with the results of Al-Zarouni et al. [52], who reported that 36% of *K. pneumoniae* isolates were ESBL-producers, while Guo et al. [53] found that 2% of *K. pneumoniae* isolates were ESBL-producers.

ESBL-producing bacteria often exhibit co-resistance to other antibiotics [30]. The detailed analysis of AMR of ESBL and non-ESBL-producing isolates to non-β-lactam AMs revealed that ESBL-producing isolates showed higher resistance to non-β-lactam AMs, as illustrated in Table 6, and this could be attributed to that plasmids carrying ESBL-encoding genes harbor AMR genes to other classes of AMs as fluoroquinolones, aminoglycosides, and sulphonamides [17, 35].

Various ESBL genes confer resistance to β-lactams in *Enterobacteriaceae*, including CTXm, TEM, and SHV, as the common ones [54]. The CXT-M gene has become the main epidemic ESBL gene worldwide [55]. In this study, 10 randomly selected *E. coli* isolates were investigated by PCR for the presence of *bla*_*CTX−M*_ and *bla*_*TEM*_ genes in addition to the *dfrA* gene, and the results were consistent with the findings of Samira et al. [29] and Youssef et al. [30]. who reported that 89.3% and 85.04% of *E. coli* isolates harbored *bla*_*CTX−M*_ and *bla*_*TEM*_ genes, respectively, while Abdel-Atty et al. [28]. found that 50% of *E. coli* isolates harbored the *dfrA* gene. The high prevalence of the investigated genes could be attributed to their presence on the plasmids that can be easily transferred among bacteria [29] and which may play a significant role in their spreading to the other species of bacteria in the consumer’s gastrointestinal tract, and that is complicated by their association together in most of the investigated *E. coli* isolates.

In this study, the coincidence rate between phenotypic and genotypic AMR in *E. coli* isolates ranged from 30% to 90% (Table 8), and there was a strong correlation between phenotypic and genotypic resistance for AMP, CFZ, FOX, SAM, and SXT, while there was a moderate correlation for CAZ, CRO, MEM, as well as TZP, and a weak correlation for FEP. These variations could be attributed to some factors, including the presence of silent genes, the presence of other expressed AMR genes than the tested ones, the resistance of β-lactams by another mechanism, such as active efflux pumps, and/or the presence of alternative mechanisms that potentially contribute to the phenotypic resistance as biofilm formation [8].

Detection of ESBL production in the laboratory can be problematic [56]. In clinical laboratories, a variety of phenotypic and genotypic assays are available for β-lactamases detection [57]. The Vitek 2 system is a fast, sensitive, and specific method for the identification and detection of ESBL-producing members of the *Enterobacteriaceae* family [31]. With the limited number of the investigated *E. coli* isolates in this study and for two genes only from the very large types of ESBL genes in consideration, the coincidence rate between ESBL production detection by Vitek 2 system and detection of *bla*_*CTX−M*_ and *bla*_*TEM*_ genes by PCR was 50% and 40% respectively and this could be attributed to several factors including the presence of silent genes, presence of other AMR genes responsible for the phenotypic resistance than the tested ones [8], the simultaneous expression for different β-lactamase genes, especially that the disagreement in this study was recorded mainly in the isolates harboring *bla*_*CTX−M*_ and *bla*_*TEM*_ genes together, outer membrane porin changes, and/or overproduction of AmpC or K1 enzyme, which may mask ESBL production [58]. Also, failure of the Vitek 2 system in detection of ESBL production could be attributed to inadequate levels of dilution or low inoculums in the isolate suspension [29], although these factors are the smallest factors that might occur in our work, where we followed the standards. In contrast to the results of this study, a high coincidence rate was recorded between ESBL production detection by the Vitek 2 system and ESBL gene detection by PCR by Sturenburg et al. [59]., while Samira et al. [29]. recorded poor and very poor agreement between them for *bla*_*CTX−M*_ and *bla*_*SHV*_ genes, respectively, and these differences could also be attributed to the usage of different Vitek cards and Vitek AES software [60].

## Conclusion

As one of the first studies on ESBL-producing *E. coli*, *K. pneumoniae*, and STEC in MPs sold in Sohag Governorate, Egypt, it increases our understanding of their epidemiology and offers significant data for future research. These MDR FBPs have been determined to be highly prevalent in MPs sold in this area, which is indicative of unsanitary handling and processing practices, fecal contamination, and possibly the presence of other enteric pathogens. As a result, MPs sold in this area are thought to be a major source of infection for consumers with these MDR pathogens and the potential for AMR genes to be transferred to the human microbial population, which could have serious public health consequences. Therefore, stricter hygiene standards must be applied immediately from farm to table, effective measures for AMR prevention and control must be created, and thorough surveillance studies of these pathogens in animals and their byproducts are recommended.

## Supplementary Information

Supplementary Material 1.

## Data Availability

Datasets generated during and/or analyzed during the current study are available from the corresponding author upon reasonable request.

## Abbreviations

MPs: Meat products
FBPs: Foodborne pathogens
*E. coli*: *Escherichia* *coli*
/* Font Definitions */ @font-face {font-family:SimSun; panose-1:2 1 6 0 3 1 1 1 1 1; mso-font-alt:宋体; mso-font-charset:134; mso-generic-font-family:auto; mso-font-pitch:variable; mso-font-signature:515 680460288 22 0 262145 0;}@font-face {font-family:SimSun; panose-1:2 1 6 0 3 1 1 1 1 1; mso-font-alt:宋体; mso-font-charset:134; mso-generic-font-family:auto; mso-font-pitch:variable; mso-font-signature:515 680460288 22 0 262145 0;}@font-face {font-family:"\@SimSun"; panose-1:2 1 6 0 3 1 1 1 1 1; mso-font-charset:134; mso-generic-font-family:auto; mso-font-pitch:variable; mso-font-signature:515 680460288 22 0 262145 0;} /* Style Definitions */ p.MsoNormal, li.MsoNormal, div.MsoNormal {mso-style-unhide:no; mso-style-qformat:yes; mso-style-parent:""; margin:0in; margin-bottom:.0001pt; text-align:right; mso-pagination:widow-orphan; direction:rtl; unicode-bidi:embed; font-size:12.0pt; font-family:"Times New Roman","serif"; mso-fareast-font-family:SimSun; mso-fareast-language:ZH-CN; mso-bidi-language:AR-EG;}.MsoChpDefault {mso-style-type:export-only; mso-default-props:yes; font-size:10.0pt; mso-ansi-font-size:10.0pt; mso-bidi-font-size:10.0pt; mso-fareast-font-family:SimSun;}@page WordSection1 {size:8.5in 11.0in; margin:1.0in 1.0in 1.0in 1.0in; mso-header-margin:.5in; mso-footer-margin:.5in; mso-paper-source:0;} div.WordSection1 {page:WordSection1;} STEC: Shiga toxin-producing *E. coli*
*K. pneumoniae*: *Klebsiella pneumoniae*
AMR: Antimicrobial resistance
ESBLs: Extended-spectrum β-lactamases
AMs: Antimicrobials
TSB: Tryptone soya broth
AES: Advanced Expert System
MDR: Multi-drug resistant
MAR: Multiple antibiotic resistance
AMP: Ampicillin
SAM: Ampicillin/Sulbactam
TZP: Piperacilllin/Tazobactam
CFZ: Cefazolin
/* Font Definitions */ @font-face {font-family:SimSun; panose-1:2 1 6 0 3 1 1 1 1 1; mso-font-alt:宋体; mso-font-charset:134; mso-generic-font-family:auto; mso-font-pitch:variable; mso-font-signature:515 680460288 22 0 262145 0;}@font-face {font-family:SimSun; panose-1:2 1 6 0 3 1 1 1 1 1; mso-font-alt:宋体; mso-font-charset:134; mso-generic-font-family:auto; mso-font-pitch:variable; mso-font-signature:515 680460288 22 0 262145 0;}@font-face {font-family:"\@SimSun"; panose-1:2 1 6 0 3 1 1 1 1 1; mso-font-charset:134; mso-generic-font-family:auto; mso-font-pitch:variable; mso-font-signature:515 680460288 22 0 262145 0;} /* Style Definitions */ p.MsoNormal, li.MsoNormal, div.MsoNormal {mso-style-unhide:no; mso-style-qformat:yes; mso-style-parent:""; margin:0in; margin-bottom:.0001pt; text-align:right; mso-pagination:widow-orphan; direction:rtl; unicode-bidi:embed; font-size:12.0pt; font-family:"Times New Roman","serif"; mso-fareast-font-family:SimSun; mso-fareast-language:ZH-CN; mso-bidi-language:AR-EG;}.MsoChpDefault {mso-style-type:export-only; mso-default-props:yes; font-size:10.0pt; mso-ansi-font-size:10.0pt; mso-bidi-font-size:10.0pt; mso-fareast-font-family:SimSun;}@page WordSection1 {size:8.5in 11.0in; margin:1.0in 1.0in 1.0in 1.0in; mso-header-margin:.5in; mso-footer-margin:.5in; mso-paper-source:0;} div.WordSection1 {page:WordSection1;} FOX: Cefoxitin
CAZ: Ceftazidime
CRO: Ceftriaxone
/* Font Definitions */ @font-face {font-family:SimSun; panose-1:2 1 6 0 3 1 1 1 1 1; mso-font-alt:宋体; mso-font-charset:134; mso-generic-font-family:auto; mso-font-pitch:variable; mso-font-signature:515 680460288 22 0 262145 0;}@font-face {font-family:SimSun; panose-1:2 1 6 0 3 1 1 1 1 1; mso-font-alt:宋体; mso-font-charset:134; mso-generic-font-family:auto; mso-font-pitch:variable; mso-font-signature:515 680460288 22 0 262145 0;}@font-face {font-family:"\@SimSun"; panose-1:2 1 6 0 3 1 1 1 1 1; mso-font-charset:134; mso-generic-font-family:auto; mso-font-pitch:variable; mso-font-signature:515 680460288 22 0 262145 0;} /* Style Definitions */ p.MsoNormal, li.MsoNormal, div.MsoNormal {mso-style-unhide:no; mso-style-qformat:yes; mso-style-parent:""; margin:0in; margin-bottom:.0001pt; text-align:right; mso-pagination:widow-orphan; direction:rtl; unicode-bidi:embed; font-size:12.0pt; font-family:"Times New Roman","serif"; mso-fareast-font-family:SimSun; mso-fareast-language:ZH-CN; mso-bidi-language:AR-EG;}.MsoChpDefault {mso-style-type:export-only; mso-default-props:yes; font-size:10.0pt; mso-ansi-font-size:10.0pt; mso-bidi-font-size:10.0pt; mso-fareast-font-family:SimSun;}@page WordSection1 {size:8.5in 11.0in; margin:1.0in 1.0in 1.0in 1.0in; mso-header-margin:.5in; mso-footer-margin:.5in; mso-paper-source:0;} div.WordSection1 {page:WordSection1;} FEP: Cefepime
MEM: Meropenem
AMK: Amikacin
GEN: Gentamicin
TOB: Tobramycin
CIP: Ciprofloxacin
LVX: Levofloxacin
NIT: Nitrofurantoin
SXT: Trimethoprim/Sulfamethoxazole
