# Supplementary material for: Prevalence of multi-drug resistant and extended-spectrum β-lactamase producing Escherichia coli and Klebsiella pneumoniae among meat products sold at Sohag Governorate, Egypt
Source: BMC Microbiol. 2025 Oct 7;25:636. doi: 10.1186/s12866-025-04392-8 (PMC12502402; doi:10.1186/s12866-025-04392-8)
Supplement: Supplementary file 1 — Supplementary Material 1. [file 12866_2025_4392_MOESM1_ESM.docx]

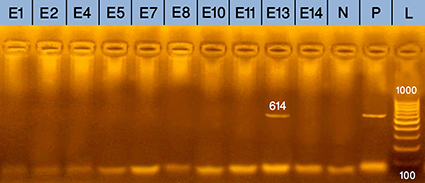


**Suppl. Fig. 1** Agarose gel electrophoresis for PCR products targeted *stx1* gene in *E. coli* isolates. Lane L: DNA ladder (100bp), lane P: Positive control, lane N: Negative control, and lanes E1-E14: DNA extracted from *E. coli* isolates showing a positive band at 614-bp in isolate number E13 only.


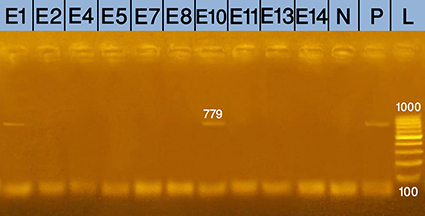


**Suppl. Fig. 2** Agarose gel electrophoresis for PCR products targeted *stx*2 gene in *E. coli* isolates. Lane L: DNA ladder (100bp), lane P: Positive control, lane N: Negative control, and lanes E1-E14: DNA extracted from *E. coli* isolates showing positive bands at 779-bp in isolates numbers E1 and E10 only.


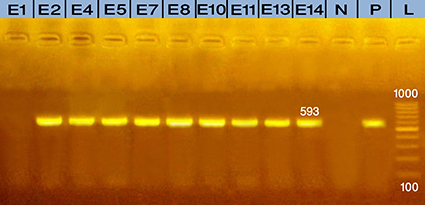


**Suppl. Fig. 3** Agarose gel electrophoresis for PCR products targeted *bla_CTX-M_* gene in *E. coli* isolates. Lane L: DNA ladder (100bp), lane P: Positive control, lane N: Negative control, and lanes E1-E14: DNA extracted from *E. coli* isolates showing positive bands at 593-bp in all isolates except isolate number E1.


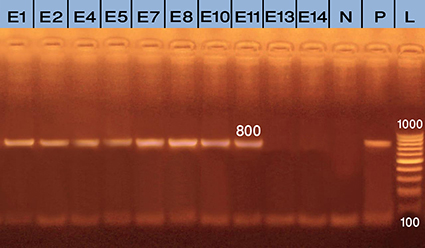


**Suppl. Fig. 4** Agarose gel electrophoresis for PCR products targeted *bla_TEM_* gene in *E. coli* isolates. Lane L: DNA ladder (100bp), lane P: Positive control, lane N: Negative control, and lanes E1-E14: DNA extracted from *E. coli* isolates showing positive bands at 800-bp in all isolates except isolates numbers E13 and E14.


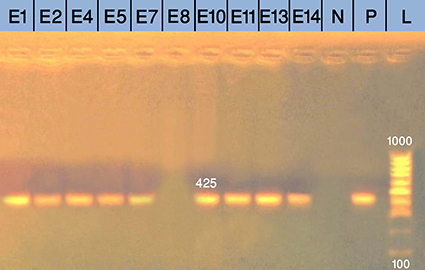


**Suppl. Fig. 5** Agarose gel electrophoresis for PCR products targeted *dfrA* gene in *E. coli* isolates. Lane L: DNA ladder (100bp), lane P: Positive control, lane N: Negative control, and lanes E1-E14: DNA extracted from *E. coli* isolates showing positive bands at 425-bp in all isolates except isolate number E8.
